# Online peer mentoring for youth with learning differences

**DOI:** 10.21203/rs.3.rs-5189431/v1

**Published:** 2024-10-23

**Authors:** Alecia Mercier, Brianna Paquette, Fumiko Hoeft, Caroline G. Richter

**Affiliations:** University of Alabama at Birmingham; University of Alabama at Birmingham; University of Connecticut; University of Alabama at Birmingham

**Keywords:** online mentoring, peer mentoring, specific learning disorder, attention deficit hyperactivity disorder, mental health

## Abstract

Youth with learning differences (e.g., specific learning disorder and attention deficit hyperactivity disorder) encounter exacerbated psychological and behavioral challenges compared to typically developing peers. Peer mentoring has proven beneficial in addressing such challenges, yet there is limited research on the efficacy of online peer mentorship programs for this population. This study investigates the effects of an online peer mentoring program on mentees’ mental health, wellbeing, and behavior across 10 outcomes: anxiety, depression, fear, cognitive challenges, behavioral concerns, executive function, sleep difficulties, self-care, interpersonal skills, and self-esteem/self-confidence. Parents retrospectively reported on their child’s mental health, wellbeing, and behavior prior to and since receiving online mentorship. Results indicated significant improvements across all outcomes following online mentorship. These findings highlight the effectiveness of online peer mentorship programs, emphasizing its potential as a valuable tool for supporting youth with learning differences.

## Introduction

### Online peer mentoring for youth with learning differences

Children with learning differences, such as specific learning disorder (SLD) and attention deficit hyperactivity disorder (ADHD), are more likely to experience academic challenges, poor mental health, and behavioral difficulties compared to typically developing children ^1^. Many of these challenges are exacerbated by the societal and psychological pressures these children face due to stigmatization and the general need to fit into an ableist society ^2^. Recent work has identified that adopting a strengths-based approach, rather than viewing learning differences as deficits that are inherently negative and need correction, may facilitate better educational, psychological, and behavioral outcomes ^3^. One promising route for encouraging children’s strengths and promoting their development is through mentorship programs ^4^.

### Students with Learning Differences

SLD and ADHD are highly prevalent learning differences, with SLD occurring in around 5% to 15% percent of children ^5^ and ADHD occurring in around 5% to 8% of children ^6^.Students with SLD face significant challenges in acquiring academic skills in areas such as reading (dyslexia), math (dyscalculia), and writing (dysgraphia). These difficulties stem from neurological differences and are not related to their educational background or intellectual abilities ^7^. SLD is frequently comorbid with ADHD, a neurodevelopmental condition characterized by ongoing inattention, hyperactivity, and impulsivity that disrupts functioning ^7,8^. Beyond academic challenges, individuals with SLD and ADHD often face higher stress, lower emotional well-being, mental health concerns, and additional social and emotional concerns ^1,9^. These individuals typically attain academic supports through the school system, but their socio-emotional needs may largely remain unmet ^4^.

### Mentorship Programs

Naturally acquired mentors have been found to mitigate the challenges often experienced by adolescents with learning differences ^10^. Evidence has shown that in-person mentoring decreases depression and improves self-esteem for youth with SLD and ADHD ^11^. Additionally, peer mentoring (i.e., pairing students with SLDs with mentors who also have SLDs) has been shown to benefit students’ academic skills and social-emotional well-being ^4^.

However, less is known about the success of mentorship programs administered virtually for individuals with learning differences. One systematic review found that online peer mentorship programs have demonstrated success at increased social communication skills and increased ability to engage in routines for children with neurodevelopmental disorders including ADHD ^12^. However, more research is needed specifically for SLD.

Further, despite differences in symptomology based on sex assigned at birth in learning differences ^13,14^, little research has examined the effects of mentoring between sexes. Limited existing literature on neurodevelopmental disorders suggests females may benefit more from online mentorship through greater responses and participation in online programs ^12^.

### Superpower Mentors

Superpower Mentors program is a novel mentorship program that connects, facilitates, and manages mentorship relationships between students or youth with learning differences (“the mentee”) and adults (“the mentor”). Mentors and mentees are paired using a near peer-mentoring method, connecting individuals who have similar background (e.g., SLD, ADHD) and lived experiences. This method of pairing mentors has been shown to facilitate the most positive outcomes in mentor/mentee relationships ^11,15^.

Consistent with prior research highlighting that mentor commitment is key to successful mentorship programs ^16^, the Superpower Mentor’s mentor selection and onboarding process is highly competitive and rigorous. Using a comprehensive framework, mentors are assessed on relatability, personal tools, drive, reliability, life story, and goals. Mentors go through a thorough vetting process with less than a 3% acceptance rate, including a resume screening, two behavioral interviews, a live-online certification course with the Superpower Mentor’s team, a criminal background check, and an interview with the prospective mentee’s parents.

The mentoring sessions are conducted online so that the mentees can attend their sessions wherever they feel most comfortable. As a result, geography and/or transportation is not a barrier to the mentor-matching process. The sessions occur once a week, and last 30–60 minutes based at a cadence most comfortable for the mentee and their family. This is the first known study to date to evaluate the effectiveness of the Superpower Mentors program.

### Current Study

A growing body of research suggests peer mentorship programs are beneficial for individuals with learning differences, however, much of the research has been conducted with children and adolescents in a school setting and limited work has evaluated the efficacy of online mentorship programs. The current study aims to address these gaps in literature by investigating the impact of an online mentorship program for youth and young adults with learning differences. Specifically, it was predicted that mentees’ mental health, wellbeing, and behavior would improve after time spent in the program. This study additionally explored whether outcomes differed by sexes.

## Methods

### Participants

Participants included 40 parents of children enrolled in the Superpower Mentors Program. Mentees included 14 females (26 males) ranging from 10 to 23 years old (*M* = 14.1 years, *SD* = 3.35). Weeks in the program working with the mentor ranged from five to 104 weeks (*M* = 26.14, *SD* = 24.07). The three diagnoses among mentees were SLD (n = 35), ADHD (n = 34), and autism spectrum disorder (ASD, n = 3). Eleven mentees had one diagnosis, twenty-eight had two diagnoses, and one had three diagnoses. Regarding comorbidities, twenty-nine participants had both SLD and ADHD, three had both SLD and ASD, and two had both ASD and ADHD. Participants primarily resided in the United States (n = 36), but two resided in the United Kingdom, one in Norway, and one did not report their country of origin. Additional sociodemographic characteristics of parents are displayed in Table 1.

### Measures

Primary caregivers retrospectively reported on ten outcomes related to their child’s mental health, well-being and ability: anxiety, depression, fear, cognitive challenges, behavioral concerns, executive function, sleep difficulties, self-care, interpersonal skills, and self-esteem/self-confidence. At the time of the study, all mentees were living with the primary caregiver that completed the surveys. Data on child outcomes prior to mentoring and since mentoring were collected retrospectively at the same timepoint. Further descriptions for each item can be seen in Table 2. Each item was rated on a 4-point scale based on the severity their child experienced each difficulty one month prior to mentoring and since completing the mentoring program: Not present (0), mild (1), moderate (2) and severe (3). Lower scores indicated fewer concerns or difficulties in that area of functioning.

## Procedures

The study was approved by the university’s Institutional Review Board (IRB#300012459), and all methods were performed in accordance with the relevant guidelines and regulations and in accordance with the Declaration of Helsinki. Participants provided informed consent prior to completing the survey. The survey was virtually administered to parents of the mentored children using Qualtrics. The survey was emailed to parents of the 118 mentees between October 2021 and August 2022, 40 of whom completed the entire survey. Parents were asked to report demographic characteristics for themselves and their child and on their child’s functioning prior to beginning the program and since the program.

### Data Analysis

Data were analyzed using IPM SPSS version 29. Descriptive statistics were evaluated for demographic characteristics and mentorship outcomes. Repeated measures analyses of variance (ANOVAs) were used to examine the effects of time, as measured by differences prior to and post-mentoring, and sex on the 10 mentorship outcomes variables (anxiety, depression, fear, cognitive challenges, behavioral concerns, executive function, sleep difficulties, self-care, interpersonal skills, and self-esteem/self-confidence). Effect size was measured by η^2^p.

## Results

**Figure 1:**
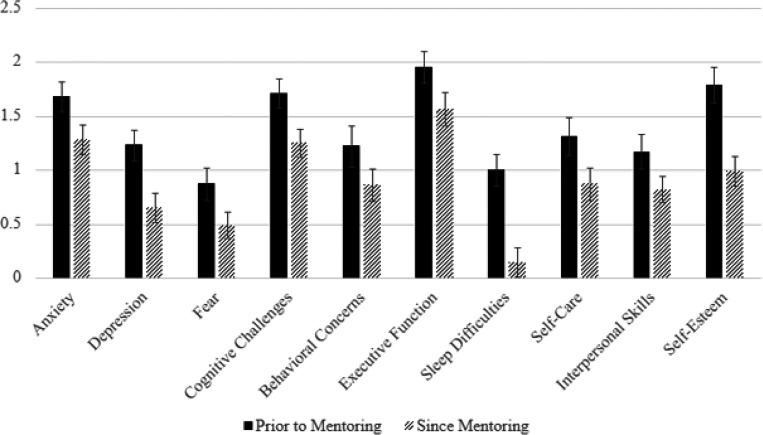
Mentee outcomes prior to and since the mentorship program *Note*. Bars represent raw scores for mental health, well-being, and behavioral outcomes. Solid Black bars represent scores prior to mentoring. Striped bars represent scores since mentoring. Error bars show standard errors.

**Figure 2:**
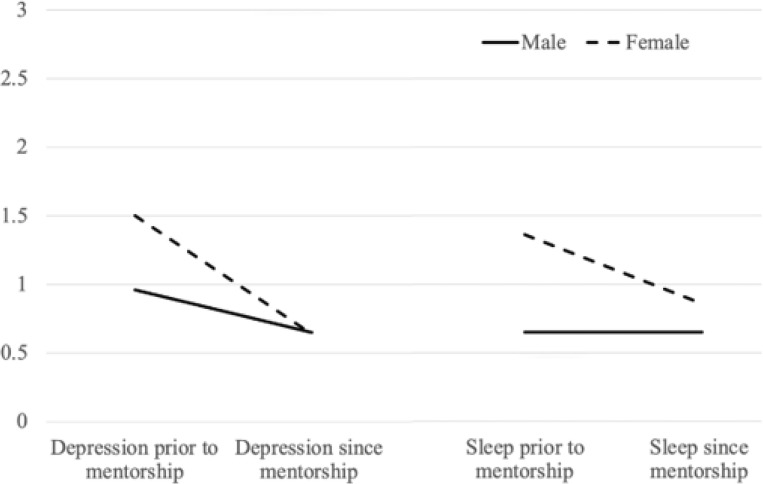
Interactions of sex by depression and sex by sleep *Note*. Lines represent change in raw scores for depression and sleep by sex. Solid lines represent males and dashed lines represent females.

Descriptive statistics for the mentorship outcomes are presented in Table 3. Two-way repeated measures ANOVAs were conducted to examine the effects of time (differences prior to and post-mentoring) and sex on mentorship outcomes. Results of the ANOVAs are displayed in Table 4. The main effect of time was significant for all mental health, well-being, and behavioral outcomes, as shown in Fig. 1. Mentees demonstrated significant improvements in anxiety, depression, fear, cognitive challenges, behavioral concerns, executive function, sleep difficulties, self-care, interpersonal skills, and self-esteem/self-confidence with effect sizes ranging from moderate to large (.13 to .52). The main effect of sex was only significant for self-esteem and behavioral concerns. Females demonstrated higher self-esteem on average and less behavioral concerns. The interaction between sex and time was significant for depression and sleep difficulties. Females demonstrated a larger decrease in both depressed mood and sleep difficulties from prior to post-mentoring compared to males. Males reported no change in sleep difficulties prior to post mentoring. These relationships are visually displayed in Fig. 2.

## Discussion

The present study examined whether an online mentorship program for youth or students with learning differences impacted mentees’ mental health, well-being and behavior. Results supported the hypotheses and mentees’ showed improvements across all outcomes: anxiety, depression, fear, cognitive challenges, behavioral concerns, executive function, sleep difficulties, self-care, interpersonal skills, and self-esteem/self-confidence.

This study supports and builds upon prior findings indicating that mentorship programs can positively benefit students with learning differences ^4,10,11^. Uniquely, this study demonstrated that mentorship programs can also be successful for students with SLD and ADHD when administered virtually (Saxena et al., 2020). Administering the programs online may help reduce potential barriers to accessing these programs, such as transportation and the availability of local mentors.

Prior work has also shown that students with learning differences benefit from having in-person peer mentors who also have learning differences ^17,18^. The current study builds upon these findings by demonstrating that peer partnerships, which tend to rely on building personal relationships between the mentor/mentee, can also be successful in an online format.

This study also explored sex differences in mentorship outcomes. Our findings suggest that sex assigned at birth plays a role in how mentees benefit from online mentorship. These results align with previous literature suggesting that females with neurodevelopmental disorders may engage more actively and benefit more from online mentorship programs due to their greater responses and participation (Saxena et al., 2020).

### Limitations

Due to the retrospective design of the study, we are unable to determine a causal relationship between participation in the mentoring program and improvement in students’ mental health, well-being, and behavior. Retrospective reports of items may be imprecise, thus leading to potential bias in the reported outcomes. Without baseline data collected prior to participation, it may not be possible to conclude the reported changes in outcomes are directly related to the mentoring program, rather than other confounding variables or placebo effects. Despite the limitations of our study, the effect sizes for the pre-post differences in anxiety, depressed mood, self-esteem, and interpersonal abilities were either larger or comparable to those reported in a previous study by Haft et al. (2019), which examined a mentorship group against two different control groups. Future studies should include prospective data collection and control groups to determine differences in the effects of mentoring programs on students with and without learning differences. Based on a recent literature review (Williams et al., 2024), only one previous study with individuals with SLD had a control group (Haft et al., 2019).

Outcomes were also assessed via parent reports rather than through self-report from the mentees, or from multiple informants. Prior studies have indicated discrepancies in self and proxy reports on various measures, often showing low to moderate inter-rater agreement ^19^. For example, parents tend to report more positive mental health and well-being outcomes for their child with a learning difference than the child reports for themselves ^20^. Thus, the positive outcomes reported by parents may not reflect the true outcomes experiences by the mentees themselves. Future research should include standardized measures completed by students, parents, and blinded observers, prior to the start of participation in the mentoring program and upon completion of the program to determine the true effectiveness of mentoring on the reported outcomes.

## Conclusion

The present study yields promising results for online mentorship experiences for students with learning differences. Overall, our findings indicate that participation in the Superpowers Mentors program is an effective approach for improving the targeted outcomes across mentees with learning differences.

## Figures and Tables

**Table 1:** Sociodemographic Characteristics of Caregivers

	n	%
Females	39	97.5
Caregivers’ race/ethnicity	40	
White and non-Hispanic	37	92.5
Black and non-Hispanic	1	2.5
Biracial and non-Hispanic	1	2.5
Other and Hispanic/Latino	1	2.5
Caregivers’ education	40	
Some college (no degree)	1	2.5
Bachelor’s degree	21	52.5
Master’s degree	17	42.5
Professional degree(PhD, MD, etc.)	1	2.5
Caregivers’ Marital Status	40	
Married	36	90.0
Divorced	2	5.0
Separated	1	2.5
Living with Significant other	1	2.5

**Table 2:** Item Descriptions of Mental Health, Well-Being and Behavior Outcomes

Outcomes	Description
Anxiety	worries, anticipation of the worst, panic attacks, nervousness, inability to relax, feelings of tension
Depression	sadness, loss of interest or lack of pleasure in hobbies, activities, or friends
Fear	of the dark, strangers, making friends, being left alone, of animals, of crowds, of school
Cognitive Challenges	difficulty concentrating, reading, writing, calculating, listening, following conversations, poor grades, negative feedback on work
Behavioral Concerns	irritability, interrupting in class, impulsivity, lack of emotional control, flexibility in thinking and adjustment of behavior
Executive Function	poor planning, organizing, time management, prioritizing, judgment, initiation of tasks
Sleep Difficulties	trouble falling or staying asleep, excessive napping, nightmares, night terrors, bed wetting
Self-Care	poor hygiene, unfulfilling activities, hobbies, or social life, inconsistent schedule, lack of time spent outdoors and/or exercising, moving, or playing sports, lack of spirituality
Interpersonal Skills	lacks trust in teachers, healthcare providers, or other adults, difficulty communicating to others about their learning challenge, trouble navigating social situations, difficulty making and maintaining friendship
Self-Esteem and Self Confidence	viewing their learning challenge as a disability, hiding their learning challenge from friends/family/teachers, lack of ability or willingness to tackle something challenging and/or advocate for themselves, viewing themselves in a negative light, believing they are incapable of getting through school

**Table 3:** Descriptive Statistics for Mental Health, Well-Being and Behavior Outcomes by Sex

	Overall (n = 40)				Female (n = 14)				Male (n = 26)			
	Pre-Mentoring		Post-Mentoring		Pre-Mentoring		Post-Mentoring		Pre-Mentoring		Post-Mentoring	
Variable	Mean	SD	Mean	SD	Mean	SD	Mean	SD	Mean	SD	Mean	SD
Anxiety	1.64	0.81	1.23	0.81	1.86	0.77	1.36	0.84	1.52	0.82	1.16	0.80
Depression	1.15	0.90	0.67	0.81	1.50	0.86	0.64	0.75	0.96	0.89	0.68	0.85
Fear	0.87	0.89	0.51	0.76	0.86	0.86	0.36	0.63	0.88	0.93	0.60	0.82
Cognitive Challenges	1.77	0.84	1.33	0.84	1.57	0.85	1.00	0.88	1.88	0.83	1.52	0.77
Behavioral Concerns	1.28	1.17	0.95	0.94	0.86	1.03	0.50	0.52	1.52	1.19	1.20	1.04
Executive Function	1.97	0.87	1.56	0.91	1.79	0.98	1.43	1.02	2.08	0.81	1.64	0.86
Sleep Difficulties	0.90	0.94	0.72	0.83	1.36	1.08	0.86	0.95	0.64	0.76	0.64	0.76
Self-Care	1.28	1.05	0.85	0.90	1.36	1.22	0.86	1.10	1.24	0.97	0.84	0.80
Interpersonal Skills	1.15	0.93	0.82	0.72	1.14	0.95	0.79	0.70	1.16	0.94	0.84	0.75
Self-Esteem and Self Confidence	1.87	1.03	1.05	0.86	1.50	1.02	0.71	0.61	2.08	1.00	1.24	0.93

## Data Availability

The data that support the findings of this study are available from the corresponding author upon request.
